# Low density lipoprotein mimics insulin action on autophagy and glucose uptake in endothelial cells

**DOI:** 10.1038/s41598-019-39559-7

**Published:** 2019-02-28

**Authors:** Lin Zhu, Guangjie Wu, Xiaoyan Yang, Xiong Jia, Juyi Li, Xiangli Bai, Wenjing Li, Ying Zhao, Ye Li, Wenzhuo Cheng, Shuli Liu, Si Jin

**Affiliations:** 1 Department of Endocrinology, Institute of geriatric medicine, Liyuan Hospital, Tongji Medical College, Huazhong University of Science and technology, Wuhan, Hubei China; 2 Department of Pharmacology, Hubei Key Laboratory of Drug Target Research and Pharmacodynamic Evaluation, School of basic medicine, Tongji Medical College, Huazhong University of Science and technology, Wuhan, Hubei China; 3 Department of Pharmacy, Tongji Hospital, Tongji Medical College, Huazhong University of Science and technology, Wuhan, Hubei China; 4 Department of Clinical Laboratory, Liyuan Hospital, Tongji Medical College, Huazhong University of Science and technology, Wuhan, Hubei China

## Abstract

Elevated plasma low density lipoprotein (LDL) is an established risk factor for cardiovascular disease. In addition to being able to cross the endothelial barrier to become accumulated in subendothelial space and thereby initiate atherosclerosis, LDL may exert a direct effect on vascular endothelial cells through activation of LDL receptor and its downstream signaling. Whether LDL can modulate the signaling for autophagy in endothelial cells is not clear. The present study firstly demonstrated that LDL can suppress endothelial autophagy through activation of the PI3K/Akt/mTOR signaling pathway and can promote glucose uptake by translocating glucose transporter 1 (GLUT1) from cytoplasm to cell membrane, actions similar to those of insulin. A co-immunoprecipitation assay found that LDL receptor (LDLR) and insulin receptor (IR) formed a complex in HUVECs. Knock down of the insulin receptor by small interfering RNA blocked the suppression of autophagy by LDL, as well as the signaling pathway involved. We conclude that LDL may mimic the action of insulin in endothelial cells, which might partly explain the increased incidence of diabetes in patients receiving some LDL-lowering therapy.

## Introduction

Low density lipoprotein is intricately involved in the atherogenic process leading to cardiovascular disease^1^. Statins, the widely prescribed cholesterol lowering drugs, reduce the morbidity and mortality of cardio- and cerebrovascular diseases and benefit billions of patients around the world^2^. However, in several clinical trials, some statins were also reported to increase HbA1c levels in patients, in addition to increasing the risk of newly diagnosed diabetes^3–7^. To date, little is known about the mechanism involved. Additional to being accumulated in the subendothelial space and initiating atherosclerosis by changing endothelial permeability^8^, LDL may exert a direct effect on vascular endothelial cells through activation of LDL receptors and downstream signaling events, *e*.*g*. cell proliferation^9^, apoptosis^10,11^ or permeability^8,12^, *etc*. However, whether LDL affects cellular autophagy remains unknown.

Autophagy is a highly conserved eukaryotic cellular process, which can deliver cytoplasmic organelles, proteins and macromolecules to lysosomes for degradation^13^. In endothelial cells, autophagy not only regulates cell survival or death, it is also involved in the modulation of a number of important cellular functions such as permeability^14,15^ and angiogenesis^16^, *etc*. Impaired autophagy in endothelial cells has been reported to play a significant role in cardiovascular diseases^17^. In the present study, we identified the effects of LDL on autophagy in endothelial cells and the intracellular signaling pathway involved, further comparing the effects of LDL with insulin, the most important molecule associated with the regulation of blood glucose homeostasis.

## Results

### LDL suppresses autophagosome formation by activation of the PI3K/Akt/mTOR pathway in HUVECs

**Figure 1:**
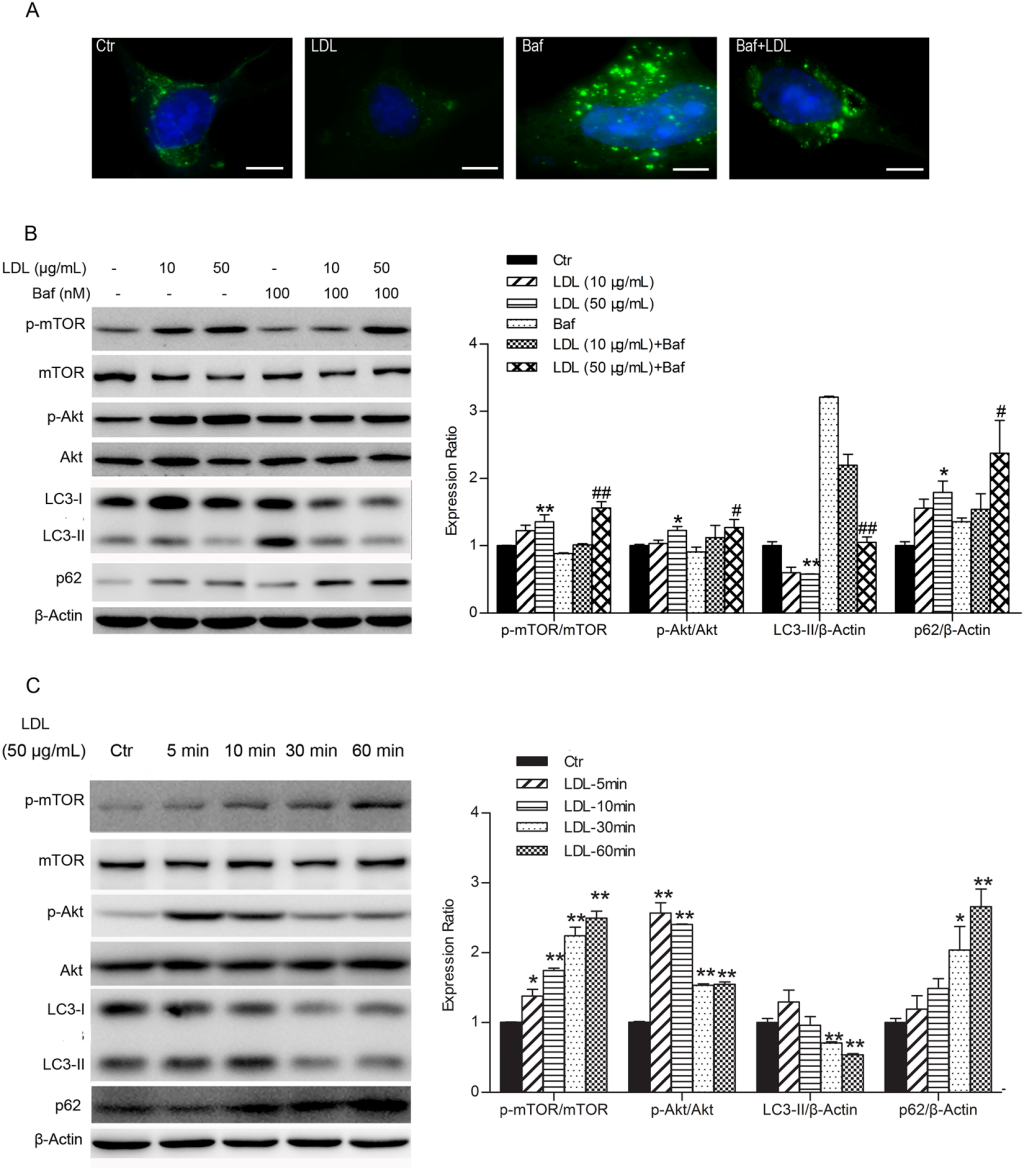
LDL suppresses autophagosome formation by activation of the PI3K/Akt/mTOR pathway in HUVECs. (**A**) HUVECs were transfected with GFP-LC3 plasmids for 48 h, then starved using serum-free medium overnight. Cells were pretreated with or without bafilomycin A1 (Baf) for 30 min and then treated with LDL (50 μg/mL) for 60 min. GFP-LC3 puncta were imaged by fluorescence microscopy. Scale bars = 10 μm, *n* = 3. (**B**) HUVECs were exposed to LDL at the indicated concentrations for 60 min with or without Baf pretreatment. Representative Western blot analysis indicating the relative expression levels of LC3-II, p62 and PI3K/Akt/mTOR pathway-related proteins. (**C**) HUVECs were treated with LDL (50 μg/mL) for the indicated time. Western blots indicating relative expression levels of LC3-II, p62 and PI3K/Akt/mTOR pathway-related proteins. The expression in control (Ctr) group cells was assigned the value of 1, *n* = 3. ^*^p < 0.05, ^**^p < 0.01 versus Ctr. ^#^*p* < 0.05, ^##^*p* < 0.01 versus Baf. Data expressed as *mean* ± *S*.*E*.*M*.

The effects of LDL on HUVEC autophagy were investigated. The number of GFP-LC3 puncta observed in HUVECs that have been transfected with GFP-LC3 plasmids indicates the content of autophagosome. As shown in Fig. 1A, incubation in LDL (50 μg/mL) for 60 min decreased the number of GFP-LC3 puncta remarkably. To explore LDL-induced autophagosome depression was due to changes in which stages of autophagy, HUVECs were pretreated with a lysosomal inhibitor (bafilomycin A1, 100 nM) which suppresses autophagosome-lysosome fusion. In this experiment, a significantly decreased quantity of LC3 puncta was also observed, suggesting that LDL decreases autophagosome formation. As shown in Fig. 1B, LDL (10 or 50 μg/mL) decreased the expression of LC3-II and increased that of p62. Furthermore, in the presence of bafilomycin, LDL enhanced the expression of p62 significantly, while the level of LC3-II expression remained suppressed, consistent with the fluorescent microscopy results. These results suggest that LDL inhibits autophagy in HUVECs via suppression of autophagosome formation rather than acceleration of autolysosome degradation.

We further explored the effect of LDL (50 μg/mL) on autophagy at different time points. As shown in Fig. 1C, LDL supressed autophagy in a time-dependent manner, peaking at the 30–60 min time point.

We further investigated the signal transduction mechanisms involved in the inhibition of autophagy by LDL. A considerable quantity of evidence suggests that the PI3K/Akt/mTOR signaling pathway is important in regulating autophagy^18,19^. As shown in Fig. 1B, LDL up-regulated the phosphorylation of mTOR (Ser2448) and Akt (Ser473), both in the absence and presence of bafilomycin. At 5, 10, 30 and 60 min after incubation in LDL (50 μg/mL), the phosphorylation of mTOR (Ser2448) increased in a time-dependent manner, peaking at the 30–60 min time point (Fig. 1C). Intriguingly, however, Akt-Ser473 was phosphorylated over a different time course, peaking after 5 min and then gradually decreasing, although remaining higher than that of the control (Fig. 1C). These results suggest that LDL might suppress autophagy in HUVECs by activation of the PI3K/AKT/mTOR signaling pathway.

### PI3K/Akt/mTOR pathway inhibitors mitigate the suppression of autophagy induced by LDL

**Figure 2:**
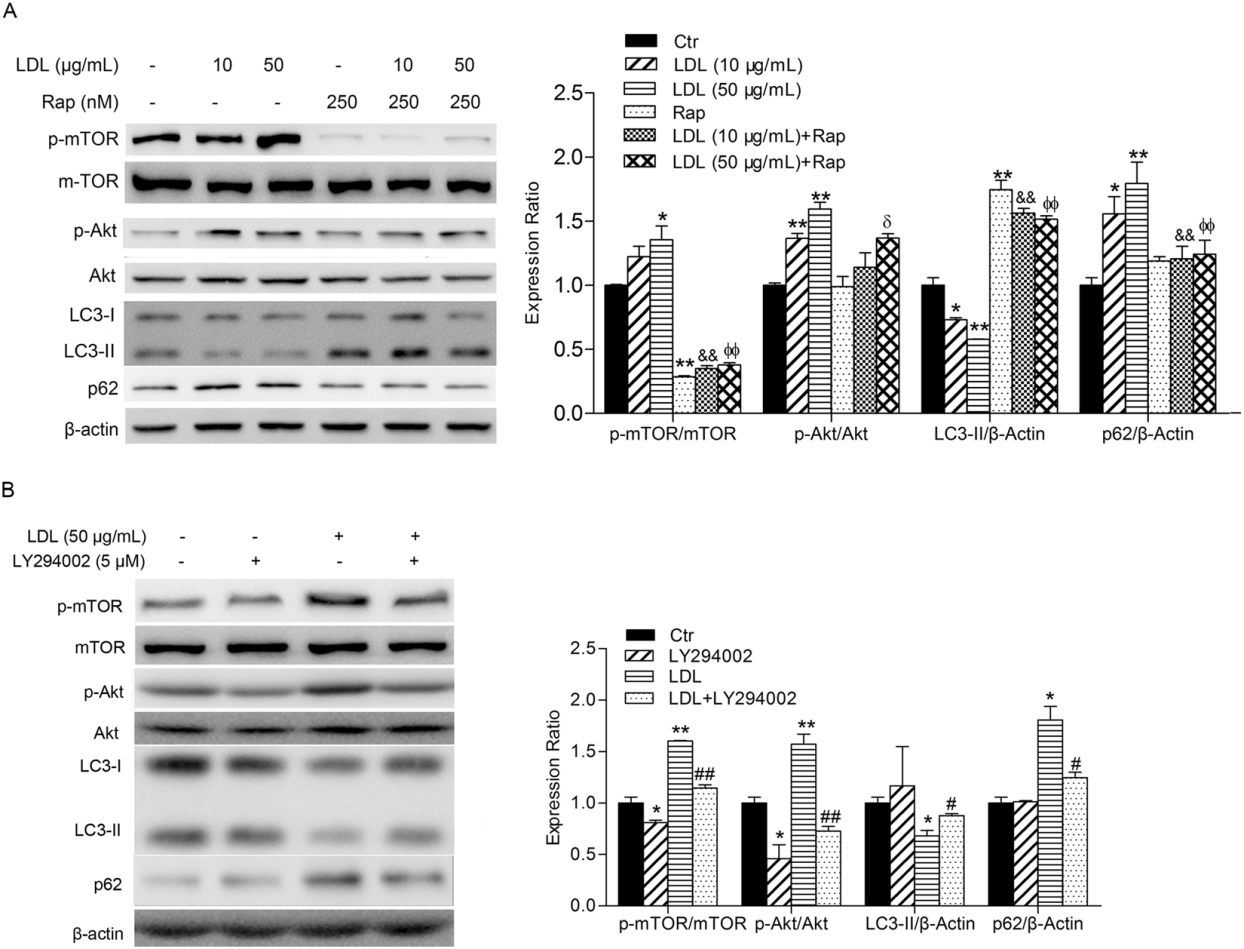
PI3K/Akt/mTOR pathway inhibitors mitigate the suppression of autophagy induced by LDL. (**A**) HUVECs were treated with LDL at the indicated concentrations for 60 min in the absence or presence of Rapamycin (Rap, 250 nM for 30 min). The expression of autophagy-associated and PI3K/Akt/mTOR pathway proteins is shown. (**B**) HUVECs were treated with PI3K inhibitor LY294002 (5 μM) for 30 min and/or LDL (50 μg/mL) for 60 min. The expression of autophagy-associated proteins and PI3K/Akt/mTOR pathway proteins was analyzed. The expression in Ctr group cells was assigned the value of 1, *n* = 3, ^*^p < 0.05, ^**^p < 0.01 versus Ctr, ^&&^*p* < 0.01 versus LDL (10 μg/mL), ^фф^*p* < 0.01 versus LDL (50 μg/mL), ^δ^*p* < 0.05 versus Rap, ^#^*p* < 0.05, ^##^*p* < 0.01 versus LDL. Data expressed as *mean* ± *S*.*E*.*M*.

To fully understand the role of the PI3K/Akt/mTOR pathway in the inhibition of autophagy by LDL in HUVECs, the effects of Rapamycin, a specific mTOR inhibitor and LY294002, a PI3K inhibitor, were analyzed. As shown in Fig. 2A, Rapamycin inhibited the phosphorylation of mTOR (Ser2448) significantly, but did not affect the phosphorylation of Akt (Ser473). The effect of LDL on autophagy was attenuated by Rapamycin, as demonstrated by a lack of significant change in LC3 and p62 expression induced by LDL following pretreatment of HUVECs with Rapamycin. LY294002 reduced the phosphorylation of mTOR (Ser2448) and Akt (Ser473) in the presence or absence of LDL and mitigated the change in LC3-II and p62 expression due to LDL treatment (Fig. 2C), demonstrating that LY294002 attenuated the suppression of autophagy caused by LDL. These results suggest that the suppression of autophagy caused by LDL in HUVECs was significantly attenuated by inhibition of the PI3K/AKT/mTOR signaling pathway, suggesting that this is the mechanism by which inhibition of autophagy is caused by LDL in HUVECs.

### LDL mimics insulin action on autophagy in HUVECs

**Figure 3:**
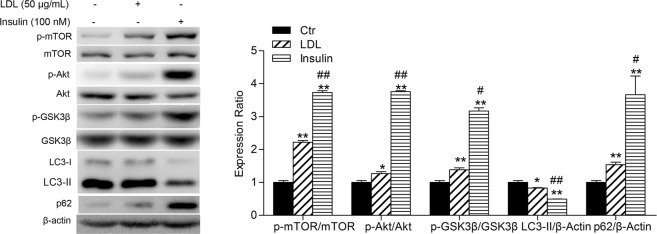
LDL mimics the role of insulin on autophagy. HUVECs were starved by culture in serum-free medium and incubated with LDL (50 μg/mL, 30 min) or insulin (100 nM, 20 min). Cell lysates were analyzed using Western blots. The expression of autophagy-associated proteins, and PI3K/Akt/mTOR and PI3K/Akt/GSK3β pathway proteins were analyzed. The expression in Ctr group cells was assigned the value of 1, *n* = 3, ^*^p < 0.05, ^**^p < 0.01 versus Ctr. ^#^*p* < 0.05, ^##^*p* < 0.01 versus LDL. Data expressed as *mean* ± *S*.*E*.*M*.

It is generally known that the PI3K/Akt pathway can be activated by activation of receptor tyrosine kinases (RTK) or G protein-coupled receptors (GPCR)^18,20^, of which IR is typical^21,22^. mTOR activation involves insulin/IGF (insulin-like growth factor) receptor-induced PI3K/Akt signaling^23^. PI3K, Akt and mTOR are positive regulators of the insulin pathway. Therefore, we further compared the effects of LDL on the PI3K/AKT/mTOR signaling pathway and autophagy with insulin in HUVECs. As shown in Fig. 3, insulin upregulated the phosphorylation of mTOR (Ser2448), Akt (Ser473) and GSK3β, in addition to activation of the PI3K/Akt/mTOR and PI3K/Akt/GSK3β signaling pathways, which were consistent with previous studies^24^. Insulin also suppressed autophagy in HUVECs, evidenced by the downregulation of the expression of LC3-II and upregulation of p62, similar to the effects of LDL. The above results suggest that LDL mimics the action of insulin on autophagy in HUVECs.

### Crosstalk between IR and LDLR in HUVECs

**Figure 4:**
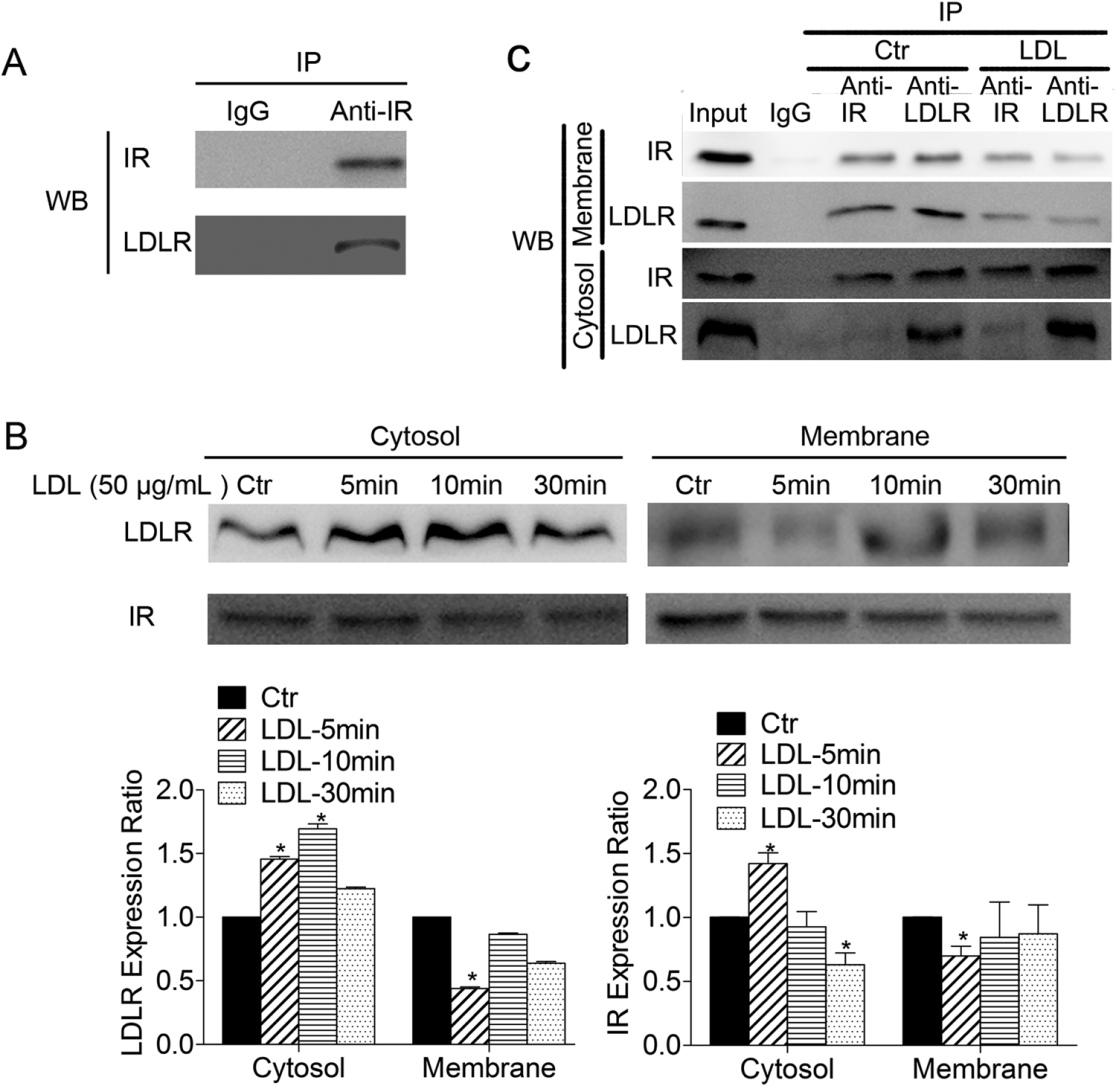
LDLR and IR form a complex and LDL stimulates endocytosis of both receptors. (**A**) HUVECs were harvested and co-immunoprecipitation was performed with IR antibody or IgG (as negative control) prior to Western blot analysis, *n* = *3*. (**B**) HUVECs were starved by culture in serum-free medium overnight then treated with LDL (50 μg/mL) for 5, 10 or 30 min. Cytoplasmic and membrane proteins were extracted and the expression of IR and LDLR analyzed by western blots. (**C**) HUVECs were starved by culture in serum-free medium overnight then treated with LDL (50 μg/mL) for 5 min. Cytoplasmic and membrane proteins were extracted and co-immunoprecipitation was performed with IR antibody, LDLR antibody or IgG (as negative control) prior to Western blot analysis, *n* = 3. Expression in Ctr group cells was assigned the value of 1, *n* = 3. ^*^p < 0.05 versus Ctr. Data expressed as *mean* ± *S*.*E*.*M*.

Both LDL and insulin operate principally through their receptors. To further elucidate the relationship between LDL and insulin, interaction between IR and LDLR was explored. As shown in Fig. 4A, immunoprecipitation by anti-IgG (as negative control) and anti-IR antibody from HUVECs resulted in bands for both LDLR and IR at their respective molecular weights. Changes in IR and LDLR after LDL treatment were further examined. As shown in Fig. 4B, the quantity of LDLR on the cell membrane decreased after LDL treatment for 5 min while LDLR levels in the cytoplasm increased. In addition, changes in IR were in line with LDLR. Moreover, the immunoprecipitation of LDLR and IR in cell membrane and cytosol was detected respectively to further illuminate the relationship between LDLR and IR (Fig. 4C). Exposing HUVECs to LDL, the protein lysates were immunoprecipitated with anti-IR antibody, we found that the quantity of LDLR was decreased on the cell membrane and increased in the cytosol, while the changes in IR were in line with LDLR after protein lysates were immunoprecipitated with anti-LDLR antibody (Fig. 4C). These results indicate that IR and LDLR probably form a complex and translocate from the cell membrane to the cytoplasm after incubation in LDL.

### IR or LDLR was required for LDL-induced autophagy inhibition

**Figure 5:**
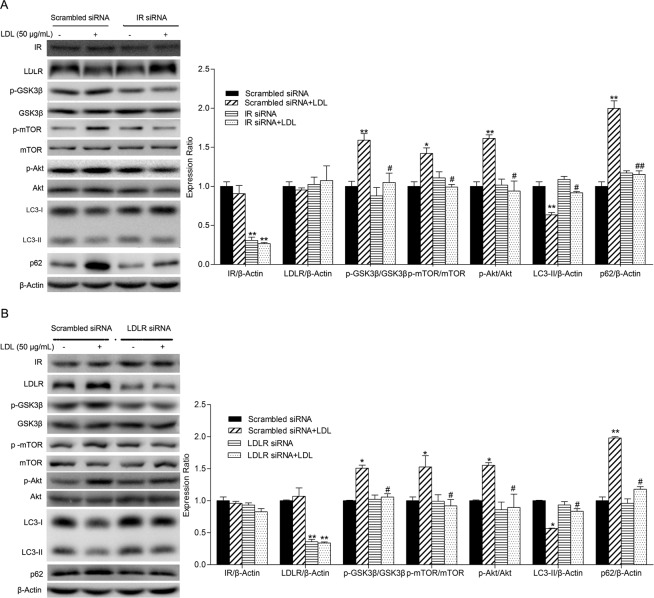
IR and LDLR knock down by siRNA attenuates autophagy inhibition by LDL. HUVECs were transfected with IR-siRNA, LDLR-siRNA or scrambled siRNA for 48 h then starved by culture in serum-free medium overnight. After successful silencing, cells were treated with or without LDL (50 μg/mL) for 60 min then cell lysates analyzed by Western blotting. The expression of IR, LDLR, autophagy-associated proteins, and PI3K/Akt/mTOR and PI3K/Akt/GSK3β pathway proteins were analyzed. The expression of Ctr group cells was assigned the value of 1, *n* = 3, ^*^p < 0.05, ^**^p < 0.01 versus scrambled siRNA alone. ^#^*p* < 0.05, ^##^*p* < 0.01 versus scrambled siRNA + LDL. Data expressed as *mean* ± *S*.*E*.*M*.

Based on the above results, we speculate that IR might mediate the suppression of autophagy induced by LDL. Autophagy was evaluated after IR and LDLR was silenced by IR-siRNA and LDLR-siRNA respectively. As shown in Fig. 5, compared to scrambled siRNA, IR-siRNA and LDLR-siRNA significantly decreased the expression of IR and LDLR, while LDLR and IR expression remained unchanged respectively. Change in LC3-II and p62 expression induced by LDL was mitigated when IR or LDLR in HUVECs was knocked down by siRNA. Furthermore, activation of the PI3K/Akt/mTOR and PI3K/Akt/GSK3β pathways induced by LDL, evidenced by elevated phosphorylation of mTOR (Ser2448), Akt (Ser473) and GSK3β, was also suppressed by siRNA.

### LDL mimics the role of insulin in the activation of glucose uptake

**Figure 6:**
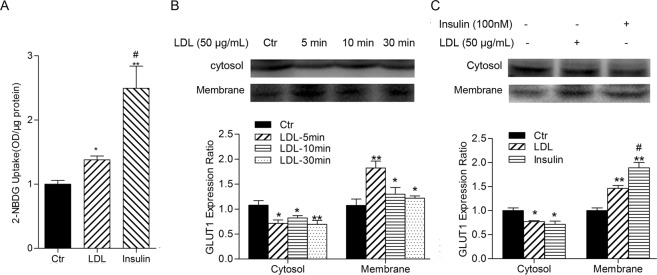
LDL mimics the role of insulin in activation of glucose uptake through translocation of GLUT1. (**A**) HUVECs were cultured in glucose-free medium prior to experimentation and incubated with 2-NBDG (50 μM) for 30 min. Cells were then incubated with LDL (50 μg/mL, 30 min) or insulin (100 nM, 20 min), washed 3 times then lysates analyzed using a microplate reader and standardized with known protein concentrations. (**B**) HUVECs were starved by culture in serum-free medium then incubated with 50 μg/mL LDL for the indicated periods. Cytoplasmic and membrane proteins were extracted then analyzed by Western blotting and GLUT1 expression quantified. (**C**) HUVECs were starved by culture in serum-free medium and incubated with LDL (50 μg/mL, 30 min) or insulin (100 nM, 20 min). Cytoplasmic and membrane proteins were extracted and analyzed by western blotting and GLUT1 expression quantified. The expression of Ctr group cells was assigned the value of 1, *n* = 3, ^*^p < 0.05, ^**^p < 0.01 versus Ctr. ^#^*p* < 0.05 versus LDL. Data expressed as *mean* ± *S*.*E*.*M*.

Since insulin is the molecule most responsible for glucose metabolism and based on the above results, we speculate that LDL may mimic the action of insulin on blood glucose regulation. 2-NBDG was used as a fluorescent indicator to measure glucose uptake by cells, principally caused by glucose transporters (GLUTs), of which GLUT1 is the major type in HUVECs^25,26^. As shown in Fig. 6A, LDL increased glucose uptake in HUVECs as did insulin, although the effect was weaker. LDL promoted GLUT1 translocation from the cytoplasm to the membrane, with optimal change observed at a treatment time of 5 min (Fig. 6B). The effect of LDL on the translocation of GLUT1 was compared with that caused by insulin. Both insulin and LDL promoted GLUT1 translocation from cytoplasm to the membrane, but the effect of LDL was weaker than that of insulin (Fig. 6C).

**Figure 7:**
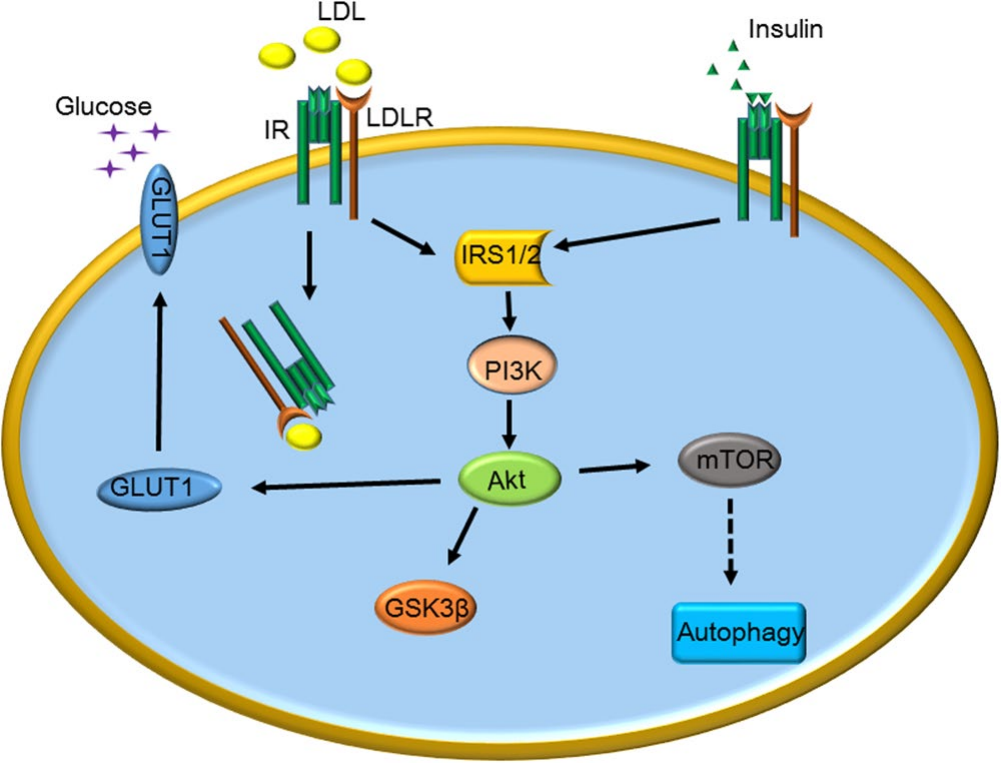
Schematic representation of the similar effects of LDL and insulin on the autophagy in HUVECs and the relationship between LDLR and IR.

Therefore, our data indicated that LDL suppresses endothelial autophagy through the PI3K/Akt/mTOR signaling pathway. Interestingly, LDLR interacts with IR and LDL mimics insulin action on autophagy and glucose uptake (Fig. 7).

## Discussion

In addition to crossing the endothelial barrier to become accumulated in the subendothelial space and initiating atherosclerosis, LDL may exert a direct effect on vascular endothelial cells through activation of LDL receptors and downstream signaling^8,9,27^. However, little is known about the effects of LDL on endothelial cell autophagy, an important process involved in human physiology, development, lifespan and a wide range of diseases including diabetes^28,29^, diabetic cardiomyopathy^30^ and atherosclerosis^30–34^. The process of autophagy is regulated by a number of autophagy-related genes (ATGs). In the initial stage of autophagy, LC3-I conjugates with phosphatidylethanolamine to form LC3-II after cleavage by ATG proteins. The quantity of LC3-II has been clearly correlated with the number of autophagosomes^35^. The receptor protein SQSTM1/p62 (sequestosome 1) targets ubiquitinated protein aggregates for lysosomal degradation and is selectively degraded via autophagy. Thus, together with LC3, it is utilized to monitor autophagic degradation/flux^36^.

Autophagy in endothelial cells may represent an important mechanism that regulates excess exogenous lipids, both native and oxidized. Ox-LDL has been shown to activate the autophagic lysosome pathway in HUVECs through the LC3/Beclin1 pathway^31^. The present study found that LDL inhibits autophagy in HUVECs, evidenced by reduced green fluorescence intensity in HUVECs transfected with GFP-LC3 plasmids and decreased expression of LC3-II, in addition to increased p62 expression. However, autophagy is not a static process and lysosomal degradation cannot be ignored. Decreased LC3 is probably an indication of a reduction in autophagosome formation or as a result of increased lysosome degradation. In order to explore the real effect of LDL on autophagy, HUVECs were pretreated with the lysosome inhibitor bafilomycin A1, demonstrating that LDL also decreased LC3 green fluorescence intensity and LC3-II expression, but increased p62 expression in the presence of bafilomycin A1. These results suggest that decreased autophagy was the result of reduced autophagosome formation and not enhanced autophagosome degradation via lysosomal turnover caused by LDL, implying that it attenuates HUVEC autophagy via suppression of autophagosome formation.

It is well accepted that the Akt/mTOR signaling pathway is a key regulator of autophagy. mTOR is a downstream target of the Akt pathway, which can promote cell growth, differentiation and autophagy^37^. We found that LDL activated the Akt/mTOR signaling pathway, delaying suppression of autophagy by LDL in relation to the sudden increase in Akt/mTOR phosphorylation. These results suggest that the Akt/mTOR pathway was an upstream modulator of autophagy induced by LDL, which may promote the formation of autophagosomes. Some differences in modification of Akt and mTOR were observed. The phosphorylation level of Akt reached its highest value when cells were treated with LDL for 5 min and then decreased in a time-dependent manner, but the phosphorylation of mTOR began to increase after 5 min and maintained the increased level, suggesting that an alternative pathway mediates the activation of mTOR by LDL, *e*.*g*. MAPK-mTOR^38,39^. Rapamycin, a specific mTOR inhibitor, attenuated the effects of LDL on autophagy in HUVECs. Generally, upregulation of Akt phosphorylation is attributed to PI3K, the upstream kinase of Akt. We observed reduced Akt activation and enhanced autophagy by LDL when HUVECs were pretreated with the PI3K inhibitor, LY294004. These results suggested that the PI3K/Akt/mTOR signaling pathway is involved in suppression of autophagy induced by LDL in HUVECs. Moreover, we found that insulin also suppressed autophagy through the PI3K/Akt/mTOR signaling pathway in HUVECs. We speculate that LDL may mimic the action of insulin on autophagy in HUVECs.

Both LDL and insulin operate through their receptors. IR and LDLR regulate glucose and lipid metabolism, which are critical in subjects with diabetes. Ramakrishnan demonstrated an intracellular co-association and plasma membrane co-localization of IR and LDLR in HepG2 cells, and insulin stimulation of the cellular expression of LDLR and enhanced its functional activity by disrupting the co-localized IR-LDLR complex^40^. Hence, we hypothesize that an interaction between LDLR and IR might occur in HUVECs.

Thus, immunoprecipitation studies were conducted and revealed that LDLR indeed forms a complex with IR in HUVECs. We found that LDLR translocated from cell membrane to cytoplasm when HUVECs were treated with LDL, which is consistent with previous reports^41^. Surprisingly, IR also displayed similar translocation patterns that did LDLR upon stimulation by LDL. It is possible that LDL may stimulate the formation of an IR-LDLR complex and subsequently promotes translocation of the complex from cell membrane to cytosol, ultimately affecting the downstream signaling pathway, including autophagy. Moreover, changes of LDLR and IR in cell membrane and cytosol before and after LDL stimulation in HUVECs, which detected by immunoprecipitation assay, further confirmed this hypothesis. However, it is not clear whether the two receptors were directly combined or co-existed in the same raft structure. As we know, upon insulin binding, IR is activated as a tyrosine-specific protein kinase and autophosphorylated which is necessary for IR to internalize. After endocytosis, the insulin and IR are dissociated. Most of the insulin is degraded, whereas the receptors are largely recycled to the cell surface^42^. Therefore, the internalization of IR induced by LDL was probably due to IR activating.

IR-specific siRNA was used to demonstrate the role of IR in the autophagy of HUVECs induced by LDL. Our results demonstrated that IR knockdown did not influence LDLR expression but decreased the phosphorylation level of Akt and its downstream molecule mTOR, therefore, the suppressed autophagy caused by LDL was aborted. These results indicate that IR mediated the inhibition of autophagy by LDL in HUVECs. And we demonstrate that the interaction of LDLR with LDL is requested to the effect of LDL on HUVECs autophagy, but not LDL per se.

Insulin is among the principal hormones that regulate glucose metabolism. Glucose uptake in cells occurs mainly through glucose transporters (GLUTs), of which GLUT1 is the major type in HUVECs^25,26^. It has been reported that insulin^43^, IL-3^44^ and NO^45^ induce the translocation of GLUTs from cytoplasm to cell membrane by activation of the PI3K/Akt signaling pathway, consequently promoting glucose uptake. Thus, we explored the effect of insulin and LDL on GLUT1 translocation and glucose uptake. We found that LDL stimulated GLUT1 translocation from cytoplasm to cell membrane and increased glucose uptake in HUVECs, which was weaker than that caused by insulin. These results strongly suggest that LDL also mimics the action of insulin on glucose uptake in HUVECs.

Statins mainly act to decrease low density lipoprotein-cholesterol (LDL-C). In fact, many alternative effects of statin have been identified beyond the lowing of LDL, including amelioration of endothelial cell function, anti-inflammatory behavior and stabilization of atherosclerotic plaques^46^. However, many meta-analyses have revealed that some statin therapy^47,48^ is linked with an increased risk of the development of diabetes mellitus in a dose-dependent manner. Compared with moderate-dose therapy, intensive-dose statin therapy exhibits a higher relative risk for developing diabetes^49–51^. The molecular mechanisms for this increased risk are complex and have not been fully elucidated. Potential mechanisms include modification to peripheral insulin signaling, exacerbated insulin resistance or impaired insulin secretion^52^. Our results indicate that LDL mimics insulin to promote the translocation of GLUT1, thereby increasing glucose uptake to achieve the effect of lowering blood glucose. Patients with or without diabetes mellitus may show increased blood glucose when LDL levels are lowered by some LDL-lowering therapies.

In conclusion, the present study provides evidence that LDL suppresses endothelial autophagy through the PI3K/Akt/mTOR signaling pathway. Interestingly, LDLR interacts with IR and LDL mimics insulin action on autophagy and glucose uptake. These novel findings may help explain the glycemic effects in patients receiving LDL lowering therapy.

## Materials and Methods

### Cell culture and treatment

HUVECs were cultured in DMEM (Hyclone, Logan, UT) containing 10% fetal bovine serum, 100 IU/mL penicillin, 100 mg/mL streptomycin and incubated at 37 °C in a humidified atmosphere containing 5% CO_2_. When confluent, cells were briefly treated (2–3 min) with 0.25% trypsin, centrifuged at 1000 rpm for 5 min, then resuspended and seeded in culture flasks or dishes for subsequent experiments. For human LDL (Yiyuan Biotechnologies, Guangzhou, China)^53,54^ treatment, When completely confluent, HUVECs were starved by culture in serum-free medium overnight and incubated with 10 μg/mL or 50 μg/mL LDL for 1 h in the presence or absence of bafilomycinA1/Rapamycin or 50 μg/mL LDL for 5, 10, 30 or 60 min. Cells were also pre-treated with LY294002 (5 μM) for 30 min prior to treatment with LDL. For insulin treatment, HUVECs were starved by culture in serum-free medium overnight and then incubated with LDL (50 μg/mL, 30 min) or insulin (100 nM, 20 min).

### GFP-LC3 plasmid transfection

HUVECs were seeded in six-well plates (Corning, USA), when 20–30% confluent, cells were transfected with GFP-LC3 plasmids for 48 h using Effectene transfection reagent (Qiagen, Hilden, Germany) according to the manufacturer’s instructions. GFP-LC3 plasmid was kindly provided by Professor Ruiguang Zhang (Union Hospital, Wuhan, China)^55^. HUVECs were then washed 3 times with PBS and starved by culture in serum-free medium overnight before treatment with 50 μg/mL LDL for 1 h in the presence or absence of bafilomycinA1. Images were obtained using fluorescence microscopy.

### Western blot analysis

HUVECs were lysed with a modified RIPA buffer (Beyotime Institute of Biotechnology, China) containing 1 mM phenylmethanesulfonyl fiuoride (PMSF). Membrane and cytosol fraction isolation was performed according to kit instructions (Proteintech, China). Protein content was determined using a Bradford assay normalized against bovine serum albumin (Sigma, USA). Protein samples were separated by SDS-PAGE gel and then electrotransferred to PVDF membranes (Millipore, USA). After incubation in blocking solution (5% non-fat dried milk, Aspen, USA), membranes were incubated over night at 4 °C with a primary antibody against β-Actin (Abbkine, Redlands, CA, USA), LC3, p62, IR, p-Akt, mTOR, p-mTOR (Cell Signaling Technology, Beverly, MA, USA), GSK3β, p-GSK3β, LDLR, Akt (Proteintech, China) used at 1:1000 dilution or GLUT1 (Cell Signaling Technology, Beverly, MA, USA) used at 1:500 dilution. Membranes were rinsed and incubated with goat anti-rabbit or goat anti-mouse secondary antibody (1:10000, Abbkine, Redlands, CA, USA) for 1 h at room temperature. Immunoreactive protein bands were developed using HRP Substrate Luminol Reagent (Millipore Corporation, Billerica, MA 01821 USA) and band intensities analyzed using a bio-Imaging system.

### Immunoprecipitation assay

Receptor protein immunoprecipitation was performed from cell lysates. Immunoblots were used to detect co-immunoprecipitation of IR and LDLR with anti-IR antibody, anti-LDLR antibody and IgG (as negative control). Each protein sample was mixed with Pierce® Protein A/G Agarose Beads (Santa Cruz Biotechnology, USA) at a ratio of 15:1 (v/v) and incubated for 15 min at 4 °C to prevent nonspecific binding then the samples were centrifuged at 14,000 rpm for 4 seconds at 4 °C and the supernatant collected. Cell supernatants were separately incubated with the specific primary antibody anti-IR, anti-LDLR or anti-IgG (Beyotime, Shanghai, China) overnight at 4 °C and then with Pierce® Protein A/G Agarose Beads for 2 h. The immunoprecipitated complexes were collected after centrifugation at 4 °C at 3000 rpm for 3 min and then extensively washed 3 times with lysis buffer, eluted with SDS loading buffer and boiled for 5 min. The samples were separated by SDS-PAGE gel and immunoblotted against anti-IR and anti-LDLR. Bands were visualized by an enhanced chemiluminescence (ECL) detection system.

### siRNA transfection

HUVECs at 60% to 70% confluence were transfected with IR-siRNA or scrambled siRNA using Hiperfect transfection reagent (Qiagen, Hilden, Germany) for 72 h according to the manufacturer’s instructions. siRNA was synthesized by Guangzhou Ribobio, China. The siRNA sequences of IR β submit and LDLR were: 5′-AAGGAGCCCAATGGTCTGATCdTdT-3′ and 5′-GGACAGAUAUCAUCAACGA-3′ respectively. Gene silencing was assessed by Western blot analysis.

### 2-NBDG uptake measurements

The 2-NBDG uptake assay was based on the incubation of mammalian cells with the fluorescent D-glucose analog 2-[N-(7-nitrobenz-2-oxa-1,3-diaz-ol-4-yl)amino]-2-deoxy-D- glucose (2-NBDG) followed by flow cytometric detection of cellular fluorescence^56^. Glucose uptake activity was measured in HUVECs using fluorescent 2-NBDG (Cayman Chem, MI, USA). HUVECs were incubated in glucose-free DMEM and subsequently with 2-NBDG at a final concentration of 50 µM for 30 min. LDL (50 µg/mL) or insulin (100 nM) was then added and incubated for 30 or 20 min respectively. Cells were lysed and fluorescence values of cell lysates measured using a fluorescence spectrophotometer (Tecan, Infinite F200PRO) using excitation and emission wavelengths of 490 nm and 520 nm, respectively. Fluorescence was normalized by total protein concentration.

### Statistical analysis

Statistical analysis was performed using GraphPad Prism version 5.0. Data were expressed as mean ± standard error of the mean (*S*.*E*.*M*). Significant differences between two groups were performed by two-tailed Student’s t test for independent variables. Differences among groups were evaluated by one-way ANOVA followed by post-hoc testing. A value of *p* < 0.05 was considered statistically significant.

## Supplementary information

Supplimentary information
