# Supplementary material for: Low density lipoprotein mimics insulin action on autophagy and glucose uptake in endothelial cells
Source: Sci Rep. 2019 Feb 28;9:3020. doi: 10.1038/s41598-019-39559-7 (PMC6395761; doi:10.1038/s41598-019-39559-7)

## Low density lipoprotein mimics insulin action on autophagy and glucose uptake in endothelial cells

Lin Zhu^1,*^, Guangjie Wu^2,3,*^, Xiaoyan Yang^2^, Xiong Jia^1^, Juyi Li^2^, Xiangli Bai^4^ , Wenjing Li^2^, Ying Zhao^1^, Ye Li^2^, Wenzhuo Cheng^1^, Shuli Liu^2^, and Si Jin^1,2^

^1^Department of Endocrinology, Institute of geriatric medicine, Liyuan Hospital, Tongji Medical College, Huazhong University of Science and technology, Wuhan, Hubei, China.

^2^Department of Pharmacology, Hubei Key Laboratory of Drug Target Research and Pharmacodynamic Evaluation, School of basic medicine, Tongji Medical College, Huazhong University of Science and technology, Wuhan, Hubei, China.

^3^Department of Pharmacy, Tongji Hospital, Tongji Medical College, Huazhong University of Science and technology, Wuhan, Hubei, China.

^4^Department of Clinical Laboratory, Liyuan Hospital, Tongji Medical College, Huazhong University of Science and technology, Wuhan, Hubei, China.

^*^These authors contributed equally to this work.

Correspondence and requests for materials should be addressed to S.J. (email: Jinsi@hust.edu.cn) .

### Supplementary information

Full-length blots used in the added Figures are as follows.

Full-length blots about the proteins exhibited in the main Figure 4C and we provide the internal reference bands for the corresponding samples.


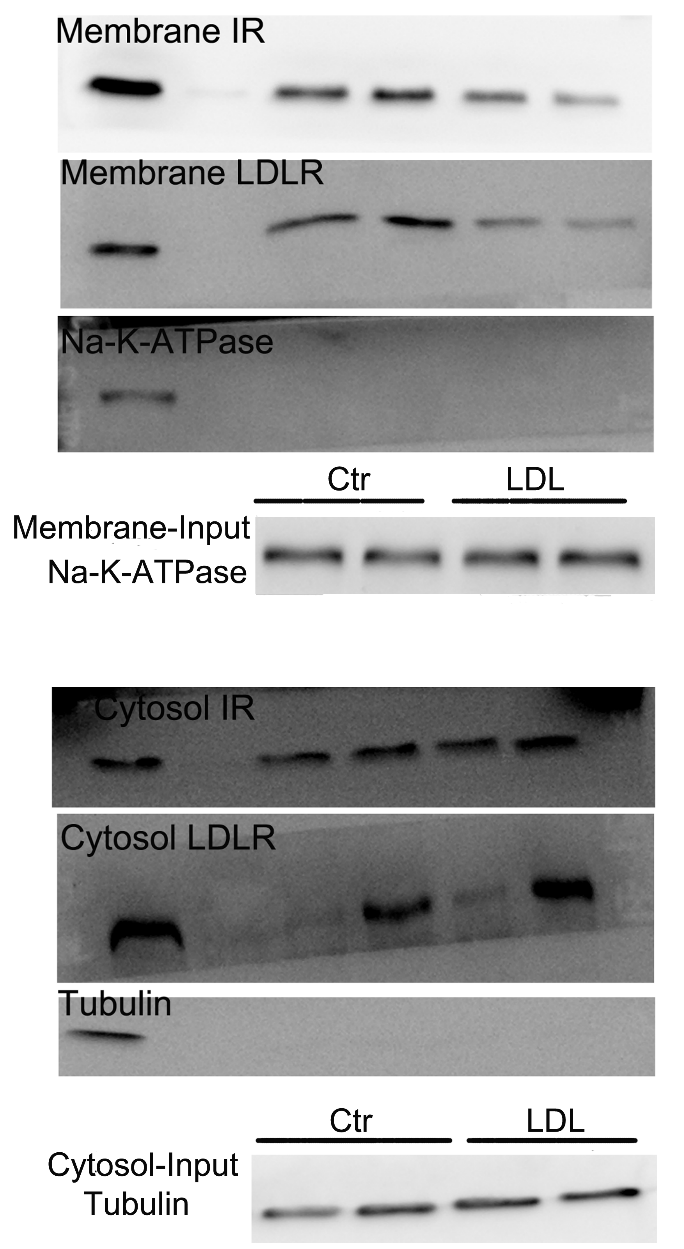


Full-length blots about some of the proteins exhibited in the main Figure 5B.


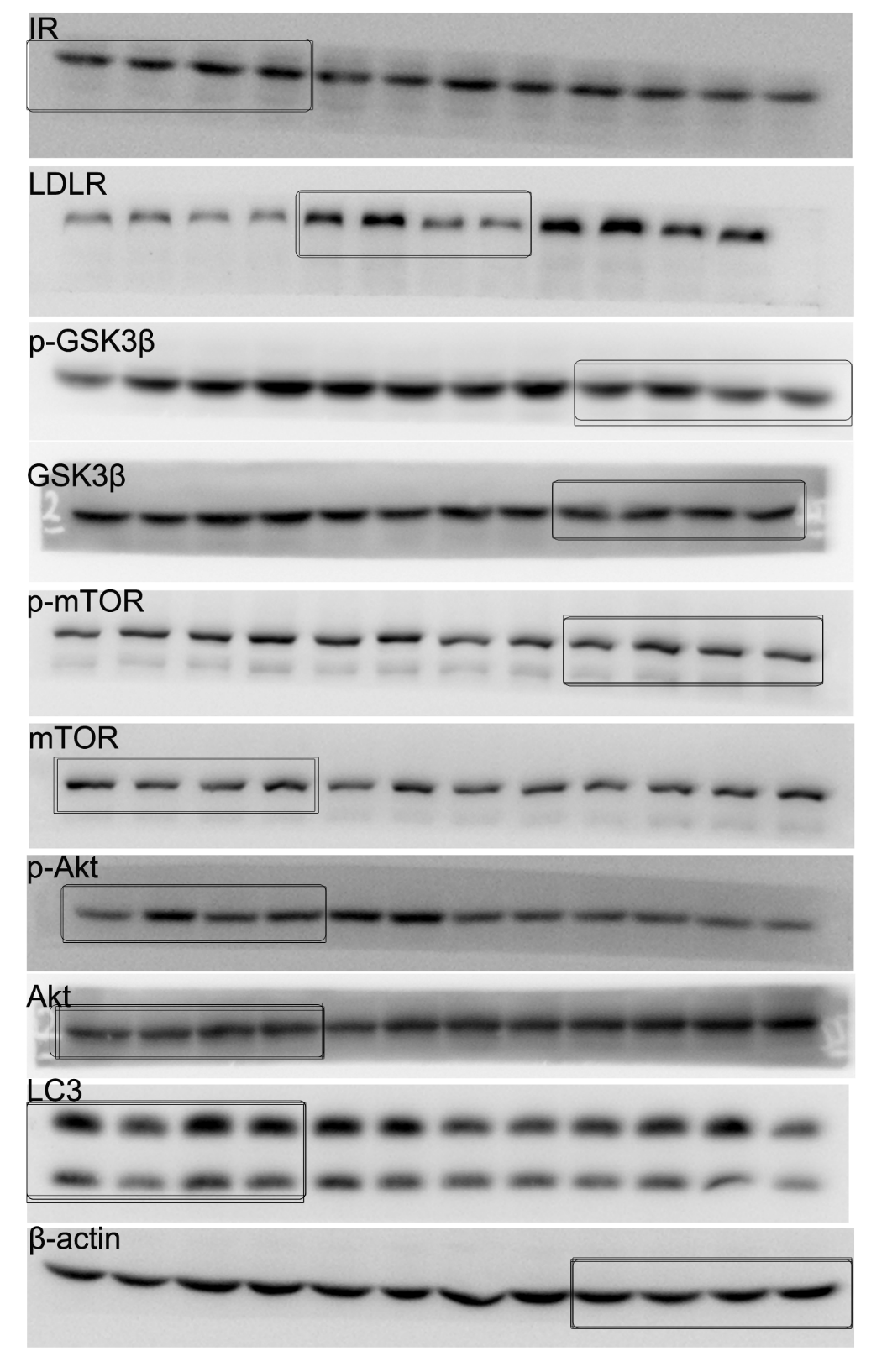

Supplement: Supplementary file 1 — Supplimentary information [file 41598_2019_39559_MOESM1_ESM.docx]
